# Disability, residential environment and social participation: factors influencing daily mobility of persons living in residential care facilities in two regions of France

**DOI:** 10.1186/s12913-017-2602-8

**Published:** 2017-09-29

**Authors:** Noémie Rapegno, Jean-François Ravaud

**Affiliations:** 1 EHESP, CNRS, Université Rennes 1, Sciences Po Rennes, ARENES, 20 avenue George Sand, 93210 La Plaine-Saint-Denis, France; 2 INSERM, CNRS, EHESS, Université Paris Descartes, CERMES3, 7 rue Guy Moquet, 94 800 Villejuif, France

**Keywords:** Disability, Residential care facilities, Social participation, (Daily) mobility, Spatial practices analysis, France

## Abstract

**Background:**

Despite the context of individualization of public policies and promotion of independent living, residential care facilities (RCFs) (called “établissements medico-sociaux” in France) still represent the main system used by disabled people. Through a study of their daily mobility, this article proposes a geographical approach to the examination of factors influencing the social participation of disabled persons with motor impairments who live in residential care facilities.

**Methods:**

The data were collected in three stages from several sources. We first carried out 24 semi-directive interviews among supervisory staff in all the institutions in two regions of France (Greater Paris and Upper Normandy) to better understand the nature of services offered by medico-social facilities. We next did field work in greater detail in 10 of these institutions. We selected residents by random sampling. These first stages then allowed us to study the mobility of residents and record their perceptions. We conducted participant observation and interviews with 81 disabled residents within the 10 RCF.

Data analysis enabled consideration not only of the role of the residential environment in people’s daily mobility, but the role of the institutions as well.

**Results:**

We identified three typical profiles of mobility practices depending on the facilities: “the islanders”, living in isolated facilities far from public transportation, or in so-called “difficult” neighborhoods; people who alternate individual and group mobility in a more or less large area; and “the navigators” who have high mobility over a very large area, often living in facilities located in urban areas. The study also enabled an analysis of the obstacles and facilitators inside and outside the residential facilities. These place restrictions on social participation by disabled adults. However, possibilities for individual negotiation may enable bypassing some obstacles.

**Conclusions:**

The three ideal-type profiles of mobility analyzed constitute adaptations to the environment by residents and the institution. The research techniques used and the presentation of data (in the form of diagrams) enabled a better understanding of the mobility of severely disabled adults living in an institution, a population that is rarely studied.

## Background

The deinstitutionalization movement and the transition to Community Living began 40 years ago in different Western countries. The rise of the Independent Living Movement coincided with the development of antipsychiatry and its fight against psychiatric institutions. Concerning residential care facilities, countries such as Norway or Sweden made the choice to deinstitutionalization as early as the 1970’s and closed their institutions for adults with disabilities in the 1990’s [1], while other countries, including France, persist in the development of institutions. However, heated debate persists, especially concerning loneliness or insecurity in community life [2]. These issues relating to of the extent of independence, control and security affect various forms of residential facilities (such as residential care homes, nursing homes, sheltered housing, etc.).

### Institutionalizing disabled persons: The French context

In a context of individualizing public policies and the promotion of independent living, residential care facilities remain numerous in France with a regular increase in their accommodation capacity. Despite encouragement at international and European level towards deinstitutionalization and the social inclusion of people with disabilities, placing disabled people in institutions remains a widely practiced solution in France. In line with the United Nations Convention for the Rights of Persons with disabilities, which recognizes (Art 19) the right to live independently and to be included in the community, the participation in society of persons with disabilities is stated as an essential principle in French legislation. However, institutions are ever more numerous. Alternative solutions, such as group homes, are still exceptional.

Residential care facilities remain a feature of the healthcare system in France (Table 1). All the services mentioned in Table 1 are under the authority of the Departmental Councils or the Regional Health Agency. Among the medico-social facilities, the residential care facilities (RCFs) for people with disabilities (or “accommodation facilities” in Table 1) provide permanent housing for disabled persons, as well as assistance for tasks of daily living, routine care, and occasional medical care. A professional team assists and accompanies people for all essential activities of daily living. The Departmental Council and/or the Regional Health Agency, depending on the accreditation obtained, pay for their operation, either on a daily basis or with a global allotment. Not being gainfully employed is a condition for residency: the RCFs included in this study accommodate disabled adults who are unable to perform professional activities in an ordinary environment, nor in a sheltered one. The services provided by these RCFs are carried out by qualified multidisciplinary teams.

**Table 1:** RCFs for people with disabilities among Health Services in France

Hospital facilities
Other treatment and prevention centers (private doctors, dispensaries, health centers…)
Other social and health establishments (laboratories, pharmacies)
Social and medico-social institutions and services (accommodation, assistance, rehabilitation)
• Institutions and services for children with disabilities
• Institutions and services for adults with disabilities
- Residential care and accommodation facilities (RCFs)
- Sheltered workshops
- Homecare services
• Institutions and services for elderly people
• Other social institutions and services
Family assistance services and institutions
Professional training institutions (staff of health and social institutions and services)

Source: Fichier national des établissements sanitaires et sociaux/National Health and Social Facilities File

In France, the development of RCFs managed by non-profit associations has long been the preferred model [3]. Home care services for disabled people benefited from legislative recognition in the 2000s, nearly 30 years after RCFs. Today, the latter still represent the principal system used by disabled persons, with twice as many persons cared for by RCFs than by home care services. Presently, 90,500 adults with disabilities live in 2800 RCFs throughout France, employing 102,900 people [4].

RCFs cover a variety of realities. Some of them accommodate less than 10 people whereas others accommodate more than 90 people in a single facility. Length of stay can vary from less than 1 year to more than 20 years [4]. Nor are facilities evenly distributed throughout France, with rural areas being over-equipped [5]. Do these disparities create differences in terms of daily mobility for residents? Moreover, are these disparities reflected in their social participation? Life in medico-social institutions is thus not uniform in nature. It is therefore especially important to explore the different lifestyles of residents conveyed by their patterns of mobility and to identify different profiles and levels of social participation.

### Disability, environment, mobility and social participation

The environment influences people’s social participation. Environmental factors can be facilitators, or on the other hand, barriers to daily mobility and people’s social participation [6]. Current theoretical models of participation, such as the International Classification of Functioning, Disability and Health (ICF) model [7] and the Disability Creation Process (DCP) model [8] include the environment as an important determinant of social participation. The ascendance of the World Health Organization’s ICF as the global standard for describing and characterizing aspects of disability has refocused attention on the role that environmental factors have in the health and participation of people with disabilities, both as individuals and as a group [9]. Foley et al. [10] found that participation in social roles for young adults with Down syndrome was considered to be influenced more by the physical environment (including public infrastructure and community organization services) than by the social environment. For example, the location of the house and the availability of transportation to and from the home are factors that may be barriers to social inclusion from the perspectives of young people.

In the area of leisure, the role of the environment is particularly significant. Taylor and Józefowicz [11] showed that accessibility and availability determine the spatial behavior of disabled persons for recreational and leisure purposes. Regardless of the location of their residence, disabled inhabitants choose forms of recreation not involving trips to the leisure areas much more frequently than non-disabled people do. They spend free time in the immediate vicinity and they stay in their home micro-area for recreational and leisure purposes. Environmental factors influence the participation of people with disabilities at the micro (individual), meso (community), and macro (societal) levels. Simplican et al. [12] also examined the attitudes and experiences of community members themselves – not just individuals with disabilities and paid care providers – for signs of social inclusion and community participation by people with intellectual and developmental disabilities. They studied community activities such as leisure activities (hobbies, arts, sports), consumption of or access to goods and services. They distinguished activities in segregated, semi-segregated and integrated settings. Segregated settings took place in group homes whereas semi-segregated settings involved paid staff or family but took place in community settings. Integrated settings were mainstream settings in the community. They also identified different levels of involvement: presence (physically being in a community with little to no contact with other people), encounters with others, and participation. Following Bigby & Wiesel [13], Simplican et al. [12] considered presence as a precursor to participation while community encounters (that is to say day-to-day interactions such as interaction with servers at a restaurant or people on a bus) are a dimension of social inclusion.

### Analyzing mobility practices

The primary aim of the study was to analyze the daily mobility and the living space of disabled adults with motor impairments living in a medico-social facility. The notion of living space overlaps with that of activity space, which refers to the combination of places frequented during daily activities and the paths connecting them [14]. Living spaces are constructed by the spatial practices of individuals and more generally by the ways of living [15]. We do not examine the nature of the activities people engage in but rather the type of mobility and the way people stake out their territory. In this article, we investigate qualitatively the daily mobility patterns of persons with disabilities in order to understand how location and location characteristics may influence social participation behavior. By studying the mobility of persons who live in RCFs, we identify places where presence and encounters are possible, whether segregated, semi-segregated or community settings. The study of mobility provides an understanding of presence and encounters in a dynamic way and can be seen as an indicator of the potential for social participation. More broadly, people’s territoriality reveals their position in geographical space, but also in social space. Several authors have studied the connection between mobility and social exclusion or well-being [16–20] especially among vulnerable populations such as immigrant women [21] or low-income populations in deprived areas [22]. The issues of mobility, and that of the accessibility of the environment, are especially important for persons with motor impairments, because of their functional limitations.

In our study of the mobility of persons with motor impairments living in medico-social facilities, we have focused on access to cultural, recreational, leisure, and sports activities [23]. Because the adults interviewed do not work, their participation in cultural and recreational activities is all the more important and thus occupies a significant place in their lives. By studying this type of mobility, we raise issues concerning the fundamental rights of disabled persons.

The issue of the location of an RCF and its impact on residents’ quality of life has been little studied in France. This article proposes a geographical approach to examine two factors that may influence people’s daily mobility and the way people construct the space they live in: the role of the residential environment (geographical isolation, transportation services, accessibility, amenities) and the role of the RCFs themselves (size, nature and regularity of activities, availability of transportation within the facilities, institutional model).

A second aim was to identify barriers to and facilitators of disabled persons’ mobility, both within the residences (linked to the life of the institution) as well as outside the residence (linked to the location of the facility, to the neighborhood). We wanted to learn to what extent the community meso environment (neighborhood businesses, transportation, etc.) as well as the organization of living accommodations influence the social participation of disabled persons. We wished to analyze the nature of people’s trips (on foot, motorized, etc.) as well as the reasons for them. We wanted to focus not only on the built environment but also on personal assistance. In particular, we examined the impact of the institutional organization more than the inter-individual relationships between professional staff and residents.

## Methods

In order to understand both the diversity of mobility practices as well as the territoriality of persons living in residential facilities for adults with motor impairments, the data were collected in three stages, from several sources.

### Two contrasting study areas: The greater Paris region and upper Normandy

The first part of the study, aiming to be exhaustive, consisted in carrying out semi-directive interviews among supervisory staff of all the institutions in these two contrasting regions of France (*n* = 24). The objective of this first step was to gain a better understanding of the nature of services offered by medico-social facilities for disabled adults with motor impairments, to describe differences in functioning between RCFs, and to obtain all necessary administrative authorizations to carry out the research. In addition, knowledge was needed on living arrangements and conditions for users. Interviews were combined with observations carried out in the facilities and their immediate environment.

The Greater Paris Region is untypical in several ways. The region is highly focused on the city of Paris and has an extensive transportation network. The region alone accounts for nearly 19% of the population of metropolitan France, making it the region with the highest population density with nearly 980 inhabitants per square kilometer. The study in Upper Normandy made it possible to introduce a comparative dimension. This region, bordering on the Greater Paris Region, is organized along the axis formed by the navigable Seine River and the freeway of the West. Cities and activities are concentrated along this backbone while the surrounding spaces have lower population density (150 inhabitants per square kilometer) and activity level, both of which increase closer to the Parisian center.

The objective of the semi-directive interviews with a member of the supervisory staff, requested in all the institutions, was to understand differences in functioning between RCFs. We also looked at the rural environment or the characteristics of the neighborhood, notably the presence of local or intermediate amenities, their accessibility, the presence of a bus stop or train station in proximity to the institution, and the presence of other people in the neighborhood. During this stage, we carried out our study in 24 RCFs, 18 of which were located in the Greater Paris Region and 6 in Upper Normandy. Only two institutions, in the Greater Paris Region, refused to meet with us.

### A sample of 10 institutions

We next did field work in greater detail in 10 of the preceding institutions, five in Upper Normandy and five in the Greater Paris Region. The selection of institutions was done taking into consideration representativeness but also the diversity of situations. In the Greater Paris Region, we selected two institutions in Paris, two near Paris, and one institution at the outer limits of the region. These 10 institutions are managed by seven different associations and were established between 1968 and 2009. They accommodate from 12 to 66 residents (Table 2). Some of these facilities have medical capabilities. We conducted additional interviews with staff members to better understand the characteristics of all residents. Depending on the institution, the average age of residents was between 28 and 45 years. In all the institutions, some residents moved about in manual wheelchairs, others in powered wheelchairs and some without technical aids.

**Table 2:** Description of the institutions in the sample

Region	Institution	Average age	Transportation services	Environment	Residents (*n*)	Persons interviewed (*n*)	Fulltime staff (*n*)
Paris region	A	35	RER STDP	Residential	30	6	45
	B	NS	Bus STDP	Business	56	9	57
	C	28	STDP	Residential	43	7	77
	D	35,5	Bus STDP	Business	12	6	17
	E	35	RER Bus STDP	Residential	13	8	20
Upper Normandy	F	45	None	Business	35	11	42
	G	45	None	Residential	66	8	NS
	H	45	None	Rural	44	9	46
	I	NS	Bus STDP	Business	47	8	56
	J	29,5	Bus STDP	Rural	22	9	24

*RER* Réseau Express Régional (suburban trains), *STDP* specialized transportation for disabled persons, *NS* not specified

### The study among residents: A necessary methodological inventiveness

After these first stages and after having selected the 10 institutions, the dual objective of the third stage was to study attitudes towards residential mobility by collecting residents’ words and perceptions – and not those of their families or of professionals working in the facility – as well as to record people’s daily movements. We used an ethnographic method combining participant observation and interviews. The observations allowed us to record people’s mobility, paths taken by the people and the obstacles encountered. The interviews complemented the observations by capturing people’s feelings and perspectives.

In order to interview residents with motor impairments and severe related disorders (cognitive disorders, language disorders), we developed an interview guide to use during the encounter. It was necessary to use several modes of communication (language easy to read and understand, pictograms), especially with persons whose command of language was limited. The aim was to gather residents’ discourse and feelings. For residents who had difficulty self-reporting, we did not collect additional data from caregivers or relatives. The interview guide was structured in four parts: the social situation of the people and their family; their history prior to institutionalization; when they enter the RCF; daily life in the RCF regarding mobility and social life.

We wished to avoid respondents being selected by the director of the institution. We thus decided to do a random sample of residents, selected using a systematic draw from the roster of residents. The aim was to have access to residents whether they had secondary disorders or not. By choosing at random, we wanted to avoid any selection bias and to have the possibility of hearing from persons who were minimally involved in the life of the facility or not very mobile and who might not have been chosen by supervisory staff because they were considered as of little interest within the framework of our research. Once the list of residents was completed, we selected 10 residents at random in each institution. The interviews yielded very different levels of information, depending on the nature of the disabilities. Sometimes people only answered our questions, without being able to explain their answers or answer all the questions (especially those with psycho-social content), whereas others gave very rich and structured replies describing their answers and their hesitations. We were thus obliged to analyze heterogeneous data, composed of highly minimalist answers (yes/no to closed-ended questions) as well as highly detailed answers to a semi-directive interview. Informed consent was sought from each potential participant. There was an 18% refusal rate. In all, we carried out interviews with 81 residents aged 21 to 70 years.

Of these 81 people, 46 were men and 35 were women. Only six were in a couple relationship (with the partner living in the same facility or not) and none of them had any dependent children. All the people had a motor impairment, often with co-occurring disorders (cognitive or language disorders).They were mostly power or manual wheelchair users. The majority had Cerebral Palsy (40 people), eight had a brain injury, four had sequelae of poliomyelitis and three were diagnosed with Spina Bifida. The others – fewer of them – suffered from a rare disease, sequelae of meningitis, or aneurysmal rupture. Most of the residents were born disabled or become disabled at an early age. Only 14 of them became disabled after the age of six. Among the residents interviewed, two were blind as well. Their length of stay within the RCF varied from a few months to 35 years. Forty-three only moved about with a power wheelchair, inside and outside, nine with a manual wheelchair, five could walk with or without technical assistance, eight were in a manual wheelchair and had to be pushed. The others used alternatively a manual wheelchair, a power wheelchair or crutches, according to their level of fatigue.

### Graphic representation of spatial practices

**Fig. 1:**
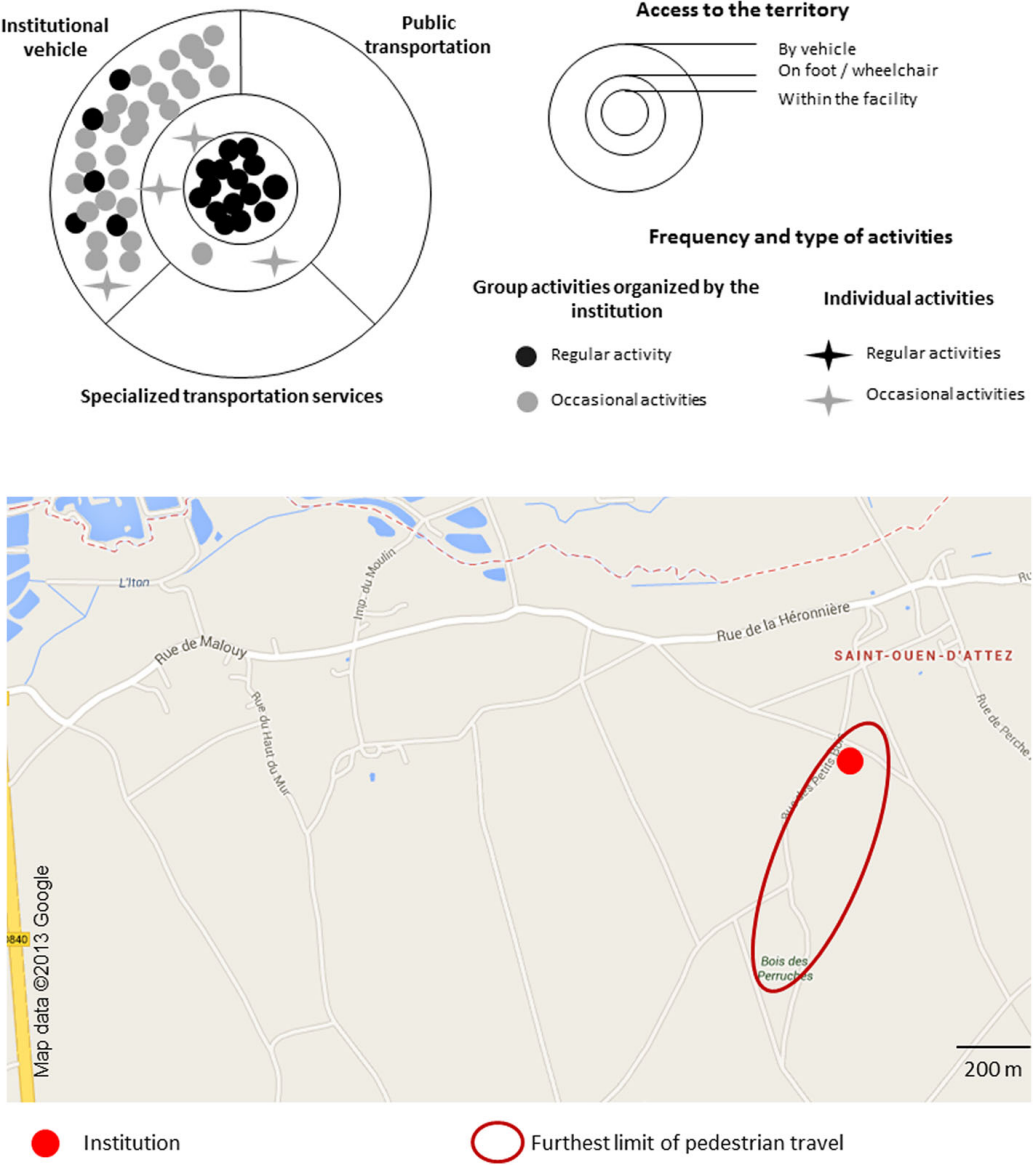
Primarily group mobility supervised by the institution

**Fig. 2:**
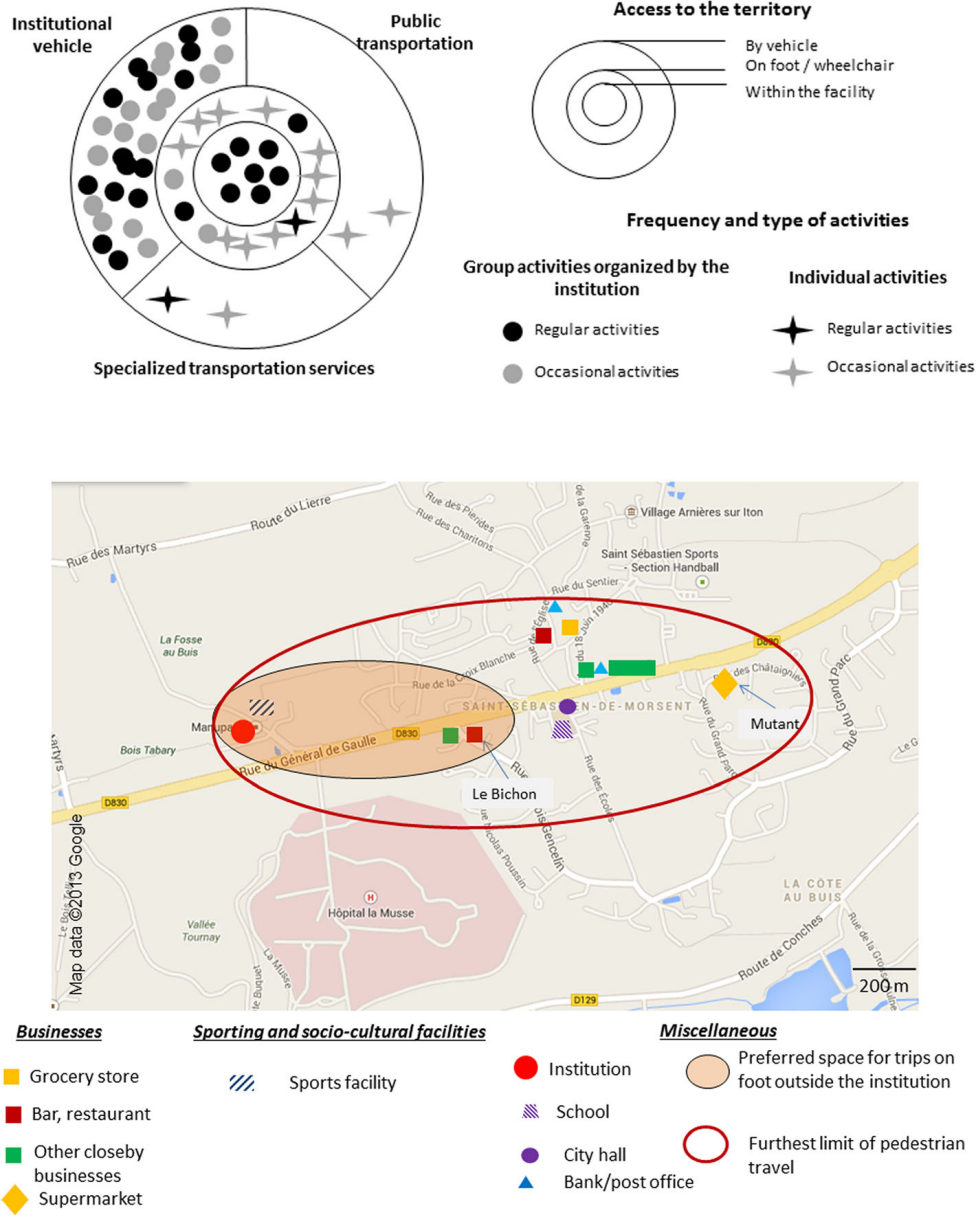
Individual and group mobility maintained by the institution within a limited environment

**Fig. 3:**
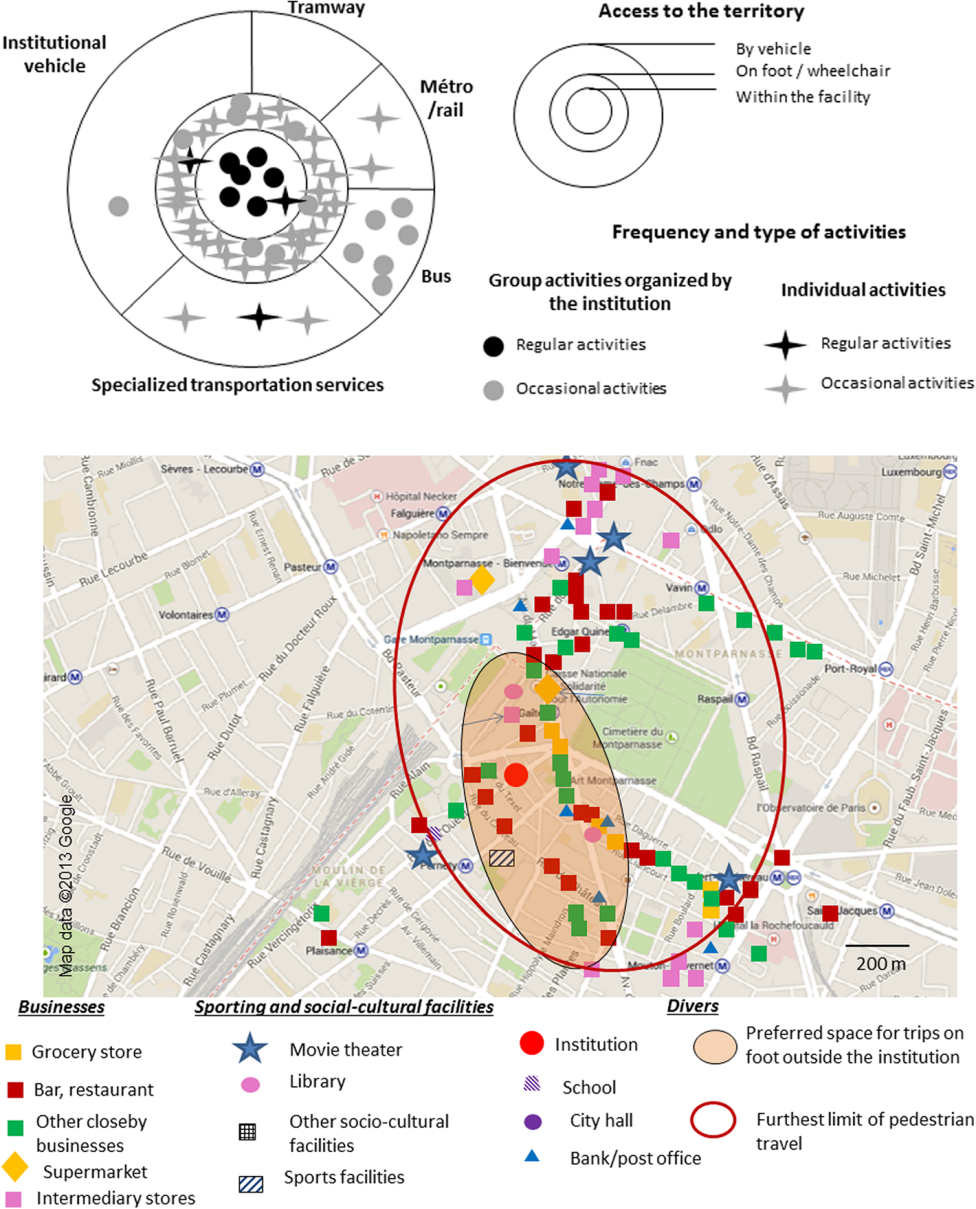
Individual mobility on several levels

Through interviews supplemented with observations, we identified all the places^1^ frequented and trips carried out by residents (individually or collectively; on foot or by public transport). We also analyzed the obstacles and the facilitators encountered by people. Interviews carried out with residents and observations done in proximity to the facility enabled us to depict people’s spatial practices in two types of illustration in each one of Figs. 1, 2, and 3.

The first illustration consists of three interlocking circles, the first representing the space of the facility, the second representing space accessible on foot or in a wheelchair, and the third representing space accessible with a vehicle or public transportation. In each figure, all activities and trips by persons in the week preceding our meeting with them are depicted – aside from trips linked to treatments or family ties. We distinguished between participation in group activities and individual trips. We also differentiated between regular outings and special trips. This first illustration informs on the type of mobility practiced by residents within the same facility, and on the relative importance of the facility in their spatial practices. This first type of scheme was based on interviews with professionals and residents.

The second illustration in the figures represents part of people’s living space, limited by pedestrian (including wheelchair users) spatial practices carried out on an individual basis. It is based on interviews with residents and on observation. We’ve used parts of maps from Google Map’s online cartography service on which we have depicted different kinds of services in proximity to the institutions, as well as the spatial practices of residents. The Google Maps areas are always to the same scale; this facilitates comparisons between different life territories. The “preferred space for trips” represents the space used by a majority of residents whereas the “outer limit of the most distant travel” (pedestrian and by wheelchair) represents the space used of a minority of highly mobile residents. When the majority of residents went out accompanied by staff, we have indicated only the extent of the most distant trips. The services depicted in the life territories were described by the participants and were frequented by some of them. We created these models for each RCF and selected the one that best illustrated each kind of mobility.

## Results: Highly diversified utilizations of space

The analysis was carried out in two stages: a first descriptive phase with the graphic representations to visualize mobility and a second phase of qualitative analysis of the interviews and of observation to analyze the obstacles encountered by people. The analysis of practices that circumscribe people’s living space revealed varied uses of this space. Among residents interviewed, all with motor impairments, it was possible to identify three very different types of mobility, from “islanders” to “navigators”, depending on the residential facility.

### An essentially group-based mobility pattern supervised by the facility

In the first configuration (Fig. 1), residents often displayed highly residence-centered personal mobility that was dependant on the facility. The majority of residents didn’t go out alone and those that went out without the help of the facility didn’t travel far. Residents made few individual trips, even near the facility in places accessible on foot or by wheelchair. They didn’t take trips using public or specialized transportation services but exclusively used the institution’s vehicle. They did not have a living space of their own. The majority of them developed an essentially similar group territoriality. They devoted little attention to their residential environment. Residents didn’t go about alone, whether in their immediate environment or in a more distant one; it was impossible for them to independently make daily trips, something that was felt by many to isolate them from any social life. This example is characteristic of the “islanders” [24, 25], those that live in relatively restricted physical and social spaces. Insularity is social and territorial.

This first profile is typical of three institutions. One of these, in the Greater Paris Region, is in a so-called “difficult” residential neighborhood, with some nearby businesses. The other two institutions, in Upper Normandy, are isolated. The site of the first Normandy institution is especially unfavorable for any individual trips. The entrance to the facility is situated on a sloping road that gives on to a curve in a departmental highway with no sidewalk or safe shoulder. The slope keeps persons in manual wheelchairs from going out alone. People in powered wheelchairs could theoretically drive there but the curve in the departmental road is considered dangerous by the institution as well as by residents and discourages any independent outings. The other Normandy institution is several kilometers distant from any businesses. Only the institution in the Paris region is served by public or specialized transportation services. Residents thus have the possibility of traveling in the entire region without needing to be accompanied by professional staff. The other institutions in Upper Normandy have neither accessible public transportation, nor specialized transportation services from outside the facility. In this case, people are dependent on the institution or on their social network. The lack of services constitutes a disadvantage in terms of social participation [26, 27].

When people do not go out alone, whatever the reason, the policy of the institution and the activities organized by the management team play an essential role in people’s social participation. Residents then have similar spatial practices, strongly inhibited by the organization of the facility.

The three institutions offer numerous regular and special activities. The organization of activities outside the facility or within the facility with volunteers assumes that the institution has developed relationships with outside organizations, professionals working in the territory or with other associations or facilities in the territory. This also assumes the presence in proximity to the facility of qualified professionals to help disabled persons. The Paris region’s facility has more opportunities than those in Normandy to manage the participation of outside organizations and to offer a richer and more varied program. These activities give a pattern to the week and constitute a busy and highly organized schedule for the residents.

Nadege, who is a particularly active resident, summarized her week:

*I don’t stop for a second. I’m in the theater group, I’m in an improvisation group (…). Theater, it’s on Monday, once every 2 weeks. Group newspaper on Tuesday, once every 2 weeks too. On Tuesday morning once every 2 weeks too we have a woman who is a retired volunteer. She reads short stories. (…) Every Wednesday morning we read a book. (…) Wednesday afternoon I attend to the menu commission with [name of professionals] and some residents. We discuss menus for the week (…). Wednesday afternoon I do gardening with an external actor, Marco. He taught us how to plant things, carrots, tomatoes. Soon we will do our vegetable garden. And soon we will work with the children too. (…) Tuesday afternoon I do a drum workshop. It’s divided into two groups. Tuesday afternoon I attend the newspaper group meeting. And Friday let’s say it’s a cool day. It’s my French teacher who comes. I like it very much. Friday, it’s pretty cool. Saturday and Sunday it’s another matter. Professionals who work at the week-end propose outings.*

Nadege had different activities every day of the week. She only carried out activities within the institution with salaried professionals or volunteers. The frequency of these activities leaves little time to develop spatial practices of one’s own.

Most people interviewed have accepted the program offered by their institution and were satisfied with the variety of leisure activities available. However, not all residents had the same tastes, a problem sometimes difficult to manage for the facilities. Some wished to have activities outside the facility while others didn’t wish to go out. All residents appeared especially desirous of activities and of individuality.

The “islander” type of mobility developed by the residents encountered is partly related to the residential environment of the facility. Most people interviewed in Upper Normandy complained of the geographical location of their facility. The remoteness from the center of town made residents dependent on the institution for any trips, whether nearby or farther afield.

### A practice of individual and group mobility supported by the facilities within a limited environment

In the second type of institution, residents combined individual and group mobility (Fig. 2). The presence of nearby services, an accessible environment and transportation serving the institution all facilitated access to individual mobility. Depending on residents’ needs and possibilities, “medico-social” professionals often accompanied them on trips, before they acquired individual mobility. Among those persons who got about alone, most had similar individual spatial practices in the immediate environment of the institution.

This type of practice is characteristic of three institutions. They are all located in residential neighborhoods where housing is the main function and where there are few nearby businesses. One is on the periphery of a large city; the other two are in peri-urban areas. During the day, these neighborhoods are relatively deserted. All these institutions are served by public transportation or by specialized transportation services.

Within these three structures, the majority of residents went out alone for various reasons and more or less distant from the institution. In the two peri-urban facilities, residents regularly went out, on foot or in wheelchair, to the closest grocery store or supermarket. This first type of place visited was primarily related to mobility with a utilitarian dimension. Residents from these two facilities also visited places more suited to social interaction, such as the closest café or bar.

The residents had good mastery of their residential environment and took advantage of all the possibilities offered, both in terms of nearby businesses and places for social interaction or promenade. They were knowledgeable about the community and had a larger living space than that of the “islanders”. However, according to them, the living space they frequented on foot or wheelchair remained very restricted. They all went to the same type of place. They moved about their residential environment to buy everyday consumer products and to maintain social relationships. For some residents, being able to move about alone was the result of a long apprenticeship. Members of the staff had the role of accompanying residents about the neighborhood, to keep them safe but also to teach them to become autonomous on their outings, when that’s possible.

Residents also had expanded individual mobility. They developed their living space individually, essentially in order to wander through the business districts. For example, it was the only outing that Christian went on. Every week at the same time, he booked a special transportation service and did some shopping. Christian and Arsène, two residents, both made the same journeys without knowing it. They withdrew money, walked a little in the mall, without always buying consumer goods, and then ate ice cream or a waffle in a fast food chain. The shopping centers constitute a place for consumption but also a strolling zone.

In the 3 RCFs, regardless of the means of transportation used, residents only went to several well-identified places. They frequented known places they were familiar with, which gave them a feeling of security; their living space was recognizable. A few people used public transportation or the specialized transportation services but most trips to more distant places were done with the institution’s vehicle.

Using a bus or a specialized transportation service did not seem to enlarge their living space compared with the space used collectively through the institution. However, it enabled them to develop individual mobility and to avoid always going out in a group, freeing them from the institutional framework. The involvement of the RCF in the organization of vehicular outings allowed residents to expand their living space and to diversify their leisure activities.

In the three facilities, residents developed their own territoriality. They appeared to have mastered the surrounding or more distant territory, whether on their own, with other residents or accompanied by a staff member. However, most of them also participated in activities organized by the institution. This enabled them to practice activities difficult to perform individually.

### Autonomous individual mobility practiced on several levels

Finally, in the third type of institution, most residents went out alone in a large area around the facility. Residents had a greater area of action. These were the “navigators”, that is *“those who live in open worlds and for whom the urban world is that of the entire city”* [24]. Most were mobile on several levels, whether in their neighborhood or in a more distant environment. They had largely taken over their neighborhood and easily moved about with no assistance from the institution. The institutional community tended to fade away.

People used different types of public transportation, individually or in a group (Fig. 3). Although residents had good individual mobility, they also participated in group outings. These were offered by the institution or organized by several residents wishing to share a moment together.

This practice predominated in four institutions, all located in the centers of towns in neighborhoods with numerous commercial streets, busy and frequented at all hours of the day. There was a broad choice both in terms of businesses and in terms of socio-cultural facilities, close by or mid distant.

Residents had good mastery of their neighborhood. They well knew the possibilities on offer and didn’t all follow the same routines. People had varied leisure activities, some regularly going to the city library, others having signed up for regular activities. Several of them went to different bars, cafés and restaurants near the institution. A multiplicity of activities was available to them. Most people had taken ownership of their residential environment and had created a social network made up of neighbors, business people and other inhabitants. This was the case for Boris:

*It can happen that I go to La Madeleine [neighborhood near the RCF]. I go with the wheelchair, it’s not too far (…). I can go to visit friends too. I have friends who live outside [the RCF]. And I go out in bars. I go to the Seven and the 13bis [name of bars]. They are next to the cinema. I have a pass card, it’s 20.50 euros per month and you can go all the time. You can go every day. But generally, we go together to the cinema with one of my friends who has the pass too.*

Residents had broad mobility; they didn’t travel the same itinerary nor go to the same places every day.

The four institutions are served by different types of transportation (bus, trains, specialized transportation). Some residents used specialized transportation but many used the bus, the tramway or the suburban train. This allowed people to be independent of the organization of the institution and group. People tried to develop their own territory, far from the institution. In general, residents demonstrated good knowledge of public transportation lines serving their institution. They mastered complex itineraries involving connecting services. The institution did not need to accompany them. They made little use of the specialized transportation service.

Their residential environment, with various leisure activities and different types of accessible public transportation, particularly encouraged social and territorial navigation. It allowed them to have a full social life as well as their own living space. People developed their own territoriality and their individuality.

In summary, residents in the 10 institutions had different modes of living as well as several types of mobility. The locality and the characteristics of the institution enabled different degrees of mobility and individual territorial practices. In all the institutions, people mainly chose their own mobility, rather than having it imposed on them, even if it had a utilitarian motive. Urban areas, for the most part, appeared to facilitate social relationships.

### Obstacles due to the environment that increase restrictions on social participation

The trend, encouraged by Disability Studies, from an individual to a social model of disability has provided a new theoretical framework for demonstrating the role of the environment. As described by the Disability Creation Process [8], the role of environmental factors is central in the causal chain explaining restrictions on social participation experienced by disabled persons. Our study shows that functional limitations are not always the main obstacle to their mobility. Regardless of the location of the institution – in the Greater Paris Region or in Upper Normandy – and the type of situation, people interviewed mentioned similar obstacles impeding their individual mobility and their social participation. People encountered difficulties at all links in the chain of movement that included the built environment, road networks, adaptation of public spaces, transportation systems and their intermodality.

In most institutions, residents complained of a lack of accessibility in their residential environment, whether for traveling around town (narrow sidewalks, absence of curb ramps, etc.) or for accessing a place open to the public (steps at the entrance to facilities). This contradicts the characteristics of a public space where everyone can mix. Residents’ mobility was constrained and limited by urban development.

Whatever the preferred means of transportation, residents encountered difficulties in their utilization. On the majority of bus lines, only one person in a wheelchair can be accommodated per bus, which prevents several disabled users from traveling together. Most difficulties in accessing buses mentioned by residents were not related to norms of accessibility but to technical and human problems. The presence of an accessible bus does not automatically mean it can be boarded. Several residents mentioned not only technical problems related to equipment – ramps that don’t descend or retract – but also the lack of goodwill on the part of bus drivers who don’t stop when they see them waiting for the bus – especially at rush hour – or baby carriages blocking access to the reserved space. It is also possible that bus drivers have not been trained to use the platforms.

Adhering to accessibility norms is not sufficient in enabling access by disabled persons. Training and consciousness-raising of bus drivers in the problems encountered by disabled persons would allow some improvement in accessibility to these means of transportation. Greater attention to specialized equipment maintenance would also reduce the frequency of technical problems. For example, an out-of-order elevator on the suburban train network results in long detours since it requires taking the train to the next station, changing platforms, taking the train back in the other direction and then taking the elevator on the opposite platform. Using public transportation considered as accessible proves to be difficult for many residents. However, the utilization of an adapted transportation service that takes one door to door also presents numerous obstacles. It requires reserving a trip long in advance and does not allow for spontaneous outings. Constraints related to the sometimes-limited hours, the restricted zone of operation of specialized transportation services, and their relatively high cost, all these factors make use of this type of transportation problematic. Although specialized transportation services sometimes make it possible for residents to extend both their spatial and social fields of activity more easily than when using ordinary public transportation, several people complained of the segregation caused by this type of transportation.

People agreed on a number of facilitating factors. The presence of close-by facilities, the nature of urban furniture, the quality of the streets (road surfacing, lowering of sidewalks, the presence of pedestrian crossings), all constituted facilitating factors for moving about the extra-residential environment. In public transportation, several things would facilitate the mobility of people we interviewed, such as improvement in signaling devices with, for example, the presence of audio signals or lighted panels indicating progress along the itinerary.

## Discussion

### Extra- and intra-residential obstacles to and facilitating factors for mobility practices

In terms of explanatory factors of people’s mobility, the extra-residential environment seemed to be more important than the intra-residential environment. In addition, the extra- as well as the intra-residential environments were more important for an understanding of mobility than people’s individual characteristics.

As in the study by Abbot and McConkey [28], it is possible to identify 4 principal barriers: lack of necessary knowledge and skills; role of support staff and service managers; location of housing; and community factors such as lack of amenities and attitudes. Among extra-residential factors, the presence or absence of amenities and the topography and accessibility of public space played an important role.

More specifically, among extra-institutional obstacles, the isolation of the RCF represented a major impediment to all individual mobility. The absence of transportation services, whether public or specialized, made residents dependant on the institution for their trips over a larger area while the absence of nearby businesses discouraged people from going out. The lack of services constitutes a disadvantage in terms of mobility and social participation. This first result is similar to numerous studies on the daily mobility of elderly persons living in suburban environments [26, 27]. Topography may also represent an obstacle, especially for people in manual wheelchairs. On the other hand, the presence of accessible public transportation (stations and vehicles), the presence of nearby equipment and their adaptation (toilets), as well as the accessibility of the environment (roadways–sidewalks, curb ramps, access ramps to buildings, elevators), all have a facilitating effect.

Among intra-residential factors, the size of the institution, the nature and frequency of proposed activities, the type of professional staff present in the institution as well as the use of vehicles belonging to the institution all constituted factors explaining mobility. At times, the number of activities offered by the institution and the high level of participation by residents at these collective occasions constituted impediments to individual outings. On the other hand, when the staff had good knowledge of the possibilities offered by the extra-residential environment and they encouraged the autonomy of residents by helping them take control of their environment, by spotting difficulties with them, by encouraging them to develop strategies for getting around these difficulties, then staff members became facilitators. Generally, the institution played a facilitating role when there were obstacles in the extra-residential environment.

### A margin for individual negotiation in getting around obstacles

The diversity of uses and perceptions of the environment illustrates the complexity of the notion of accessibility and the difficulty of constructing an urban environment for everyone. Respecting legal criteria that define minimum requirements is insufficient in allowing each person to take possession of his or her environment.

People can sometimes adapt their itinerary according to known obstacles, and work out tactics for daily living [29, 30]. Faced with perceived obstacles, residents found strategies for moving about in their residential environment by making detours, by taking other sidewalks or by using the road. When possible, some residents preferred to expand their living space in order to gain access to a business without steps. However, using this technique necessitates having many possibilities in one’s neighborhood, which was not the case for most residents. Only those residents in institutions located in business districts could use this strategy and choose the business that suited them. Faced with all these obstacles, often perceived as dangerous, some people preferred to limit their movements and their living space by only going out when accompanied by institutional staff. The development of everyday strategies requires very good knowledge of the residential environment. Indeed, this assumes one has recognized the obstacles and then developed new itineraries in order to avoid these impediments. Problems encountered by residents in moving about in public spaces – spaces for meeting people and that encourage social interaction [31] – are proof of the difficulty disabled person have in gaining access to participation in social life.

The perception people have of their extra-residential environment and the manner in which they have taken possession of their neighborhood can be a factor in explaining their mobility as well. Residents were little satisfied by the location of the RCF in a rural community, on the outskirts of a town center or in a residential neighborhood with few businesses and composed of people who commute between the place they live and the place they work, whether or not the neighborhood is posh or the object of urban policy planning. In these cases, their living space was often reduced. The “islander” type of mobility developed by residents was partly related to the way residents perceived this environment. Residents moved about less in neighborhoods that were not busy or animated.

In order to follow up this study, it would be interesting to analyze the role of the other actors, in particular the social and family networks that are involved in residents’ mobility. Knowing the frequency and nature of these interventions would lead to a better understanding of the links between these actors and enable the identification of other ways of adapting to the extra- and intra-institutional environment. This would allow refining the three types of profiles described. It would also be interesting to examine whether the three types of profiles are valid for mobility related to treatments. Finally, taking into account virtual space and the way people utilize and inhabit this space would enable integrating other dimensions into each type of profile.

### Limitations of the study

Geographical location is insufficient in explaining the diversity of recourse to the territory around the institutions. Depending on their policies and characteristics (number of people accommodated, type of staff, number of vehicles, etc.), staff members have different relationships with their territory and use territorial resources differently. The same thing applies to residents’ individual mobility. Their length of time in the institution, their age, their mode of travel (manual or powered wheelchair, on foot) or even their socio-cultural background, all these are factors that influence the construction of the living space. The very personal perception and ownership of the neighborhood plays a role in mobility and participation in everyday activities [32, 33]. Social fears of residents, including fear of social rejection and fear of losing valued aspects of identity can also constitute impediments to social participation [34].

In the same way, several variables known to influence mobility have to be taken into account. Focusing on characteristics of institutions, particularly their locations, tends to minimize the importance of individual characteristics in understanding the different types of mobility. Individual factors such as age, type of disability, and mode of transportation (wheelchair users or not) also play an important role in explaining differences in mobility, especially concerning public transportation use [35, 36]. In our study, regardless of the institution, some people could not go out alone because of disorders associated with motor impairments as well as visual impairments, problems of spatial orientation or depth perception, or cognitive disorders. Often, disorders associated with their motor impairment prevented them from moving about safely. Users had different ways of responding to these difficulties. Some people preferred mutual aid and solicited other residents while others asked to be accompanied by professional staff. In spite of an apparently welcoming environment, some residents could not go out alone and depended on professional staff from the institution for their trips. In this case, the activities organized by the institution were essential and constituted the only opportunity and possibility of going out.

Furthermore, environmental barriers that can be overcome are not absolute barriers [37]. It is essential to take into account human support. Many of the skills necessary to becoming familiar with the environment are acquired with practice [13]. The physical and social environment can be modified, but residents might also benefit from training in how to get on and off public transportation or in the use of their mobility devices. As Bigby & Wiesel [13] demonstrated, this understanding can shift responsibility for social inclusion of people with disabilities from just the individual or disability services to the characteristics of the environment in localities. People’s impairments can influence their participation. However, social participation has many determinants and for a complete understanding of disability, it is also necessary to take environmental factors into account.

In spite of the precautions we took to respect the diversity of the situations observed, it is not possible to extrapolate to finer subcategories of the population in terms of age, sex, type of impairment, means of locomotion or time living in the facility. While these variables influence mobility, the methodology of this study does not enable such an analysis with the necessary degree of accuracy.

The patterns noted result from an initial examination of residents’ mobility. They indicate places where presence and encounters are possible but they do not enable evaluating a sense of membership, belonging or involvement of people. The study does not concern people’s satisfaction or measure their quality of life. It sheds light on the social inclusion of RCF residents by taking into account disabled peoples’ spatial practices.

## Conclusions

The research techniques used enabled a better understanding of the mobility of severely disabled adults living in an institution, a population that is rarely studied. The illustration of patterns of mobility for each RCF enabled comparison of different situations as well. This method can be used in other contexts (persons living at home, in alternative housing, in France and abroad). It is also important to take into account not only the obstacles, as the majority of articles do, but also things that facilitate mobility. The three described profiles represent three types of mobility. They are neither profiles of medico-social institutions nor profiles of people. Indeed, the constructed typology is not an a priori theoretical typology of the institutions, but rather a practical typology of institutions as they are used by residents, from the perspective of their mobility within the framework of their leisure activities. We have drawn up the dominant mobility practices within each institution but exceptions may exist. Within each RCF, there may be residents whose behavior runs counter to that of the majority. These profiles are multifactorial. They are the result of the interaction – in the ICF sense – between intra- and extra-residential factors and individual behaviors.

Strategies put in place by the institution as well as by those persons concerned often constitute a solution to obstacles in the extra-residential environment. The three ideal-type profiles of mobility analyzed constitute adaptations to the environment by residents and the institution. The profiles were often the result of negotiations concerning the extra-residential environment in order to meet people’s mobility and participation needs. The “islander” model constitutes a type of response given by an institution in relation to its environment. At the extra-residential level, some institutions are located in more facilitating environments than others are. This is often the case in the third type of situation. Within the residence, institutions adopted strategies to compensate for obstacles in the extra-residential environment. A space for negotiation was thus possible and different strategies could be adopted.

In light of this study, we can make several recommendations. For existing institutions, it is necessary to develop policies that encourage or offer incentives to promote the development of close-by and accessible public transportation. If this is not possible, care must be taken to ensure that practical transportation alternatives meet actual needs. For example, funding could integrate a specific financial envelope aimed at developing specialized transportation for the RCF, to compensate for a lack of public transportation facilities.

Little thought has been given to the location of RCFs in terms of mobility and the social participation of people. Economic opportunities and constraints encountered in the creation of an RCF are key factors in explaining its location. Since 2009, the development of RCFs is regulated in France by the Regional Health Agency. Today, it is mainly the internal characteristics of the institutions that are examined and evaluated during the authorization procedure. However, it appears essential to introduce a geographical criterion concerning the location and isolation of the RCFs by assessing the quality of the residential environment in terms of accessibility and the density of public services and businesses.

This “optimal” location, close to local shops, public transportation and sports and socio-cultural facilities, in an accessible environment, is just as valid for people with disabilities living in the community as for others. It is thus necessary to integrate these issues into urban policy and the training of social housing landlords, real estate promoters and development planners.

## Abbreviations

DCP: Disability creation process
ICF: International Classification of Functioning, Disability and Health
RCF: Residential care facility
RER: Réseau express régional (suburban trains)
STDP: Specialized transportation for disabled persons

## Footnotes

1 Shops (supermarkets and small shops), services and facilities (common services such as banks, hairdressers or post offices), leisure (sport, culture ...), associative or religious activities, outings (cafe, restaurant ...).
